# Hypothermic oxygenated perfusion inhibits HECTD3-mediated TRAF3 polyubiquitination to alleviate DCD liver ischemia-reperfusion injury

**DOI:** 10.1038/s41419-021-03493-2

**Published:** 2021-02-24

**Authors:** Wei Zhou, Zibiao Zhong, Danni Lin, Zhongzhong Liu, Qiuyan Zhang, Haoyang Xia, Sheng Peng, Anxiong Liu, Zhongshan Lu, Yanfeng Wang, Shaojun Ye, Qifa Ye

**Affiliations:** 1 Zhongnan Hospital of Wuhan University, Institute of Hepatobiliary Diseases of Wuhan University, Transplant Center of Wuhan University, Hubei Key Laboratory of Medical Technology on Transplantation, Engineering Research Center of Natural Polymer-based Medical Materials in Hubei Province, Wuhan, China; 2 The First Affiliated Hospital, Zhejiang University School of Medicine, Department of Hepatobiliary and Pancreatic Surgery, Zhejiang Provincial Key Laboratory of Pancreatic Disease, Innovation Center for the Study of Pancreatic Diseases, Hangzhou, China; 3 The 3rd Xiangya Hospital of Central South University, Research Center of National Health Ministry on Transplantation Medicine Engineering and Technology, Changsha, China

**Keywords:** Molecular biology, Diseases

## Abstract

Ischemia-reperfusion injury (IRI) is an inevitable and serious clinical problem in donations after heart death (DCD) liver transplantation. Excessive sterile inflammation plays a fateful role in liver IRI. Hypothermic oxygenated perfusion (HOPE), as an emerging organ preservation technology, has a better preservation effect than cold storage (CS) for reducing liver IRI, in which regulating inflammation is one of the main mechanisms. HECTD3, a new E3 ubiquitin ligase, and TRAF3 have an essential role in inflammation. However, little is known about HECTD3 and TRAF3 in HOPE-regulated liver IRI. Here, we aimed to investigate the effects of HOPE on liver IRI in a DCD rat model and explore the roles of HECTD3 and TRAF3 in its pathogenesis. We found that HOPE significantly improved liver damage, including hepatocyte and liver sinusoidal endothelial cell injury, and reduced DCD liver inflammation. Mechanistically, both the DOC and HECT domains of HECTD3 directly interacted with TRAF3, and the catalytic Cys (C832) in the HECT domain promoted the K63-linked polyubiquitination of TRAF3 at Lys138. Further, the ubiquitinated TRAF3 at Lys138 increased oxidative stress and activated the NF-κB inflammation pathway to induce liver IRI in BRL-3A cells under hypoxia/reoxygenation conditions. Finally, we confirmed that the expression of HECTD3 and TRAF3 was obviously increased in human DCD liver transplantation specimens. Overall, these findings demonstrated that HOPE can protect against DCD liver transplantation-induced-liver IRI by reducing inflammation via HECTD3-mediated TRAF3 K63-linked polyubiquitination. Therefore, HOPE regulating the HECTD3/TRAF3 pathway is a novel target for improving IRI in DCD liver transplantation.

## Introduction

Owing to the shortage of donor organs, marginal donor allografts are increasing becoming the main source of grafts^1^. Particularly, liver allografts from donations after cardiac death (DCD) may increase the pool of organs by as much as 20%^2^. Unfortunately, warm ischemia, cold ischemia, and subsequent reperfusion phases during DCD liver transplantation can lead to inevitable ischemia-reperfusion injury (IRI), which results in delayed graft dysfunction and ultimately decreases long-term graft survival^3–5^. The ischemic phase can activate innate immune cells to initiate the tissue repair process required to restore homeostasis and provide defense against microbial invasion. However, in the subsequent reperfusion phase, the excessive activation of immune responses causes oxidative stress and, local and systemic inflammation, thereby aggravating liver damage^6,7^. To alleviate IRI, dynamic preservation technology for grafts has been developed. Specifically, hypothermic oxygenated perfusion (HOPE) has been demonstrated to decrease oxidative stress and cellular inflammation in DCD liver transplantation^1,8–10^. However, little is known about its mechanism of action.

Ubiquitin modification plays an indispensable role in regulating inflammation and immune responses^11^. Ubiquitin-protein ligases (E3s) are commonly divided into two types: HECT and RING^12^. HECT ligases comprise 28 members grouped into three subfamilies^12^. The homologous to the E6-associated protein carboxyl terminus domain containing 3 (HECTD3), a member of the third subfamily of HECT ligases, is strongly expressed in the human liver^13^. HECTD3 consists of 861 amino acid residues, with a DOC domain (219–397) at the N-terminus and a HECT domain (512–857) at the C-terminus^14^. After a specific substrate combines with the DOC domain, it is usually ubiquitinated and modified via a ubiquitin-HECT thioester complex^11,14^, thereby exerting its biological activities. Itch, a HECT E3 ubiquitin ligase, promotes the degradation of RORγt via K48-linked polyubiquitination to suppress colonic inflammation^15^. In addition, HECTD3 promotes nondegradative K27-linked and K29-linked polyubiquitination of Malt1 at K648 and K27-linked polyubiquitination of Stat3 at K180 to activate the pathogenic Th17 lineage, thereby aggravating the severity of experimental autoimmune encephalomyelitis (EAE) in mice^16^. These results show that HECTD3 plays a vital role in coordinating inflammation and immune responses via ubiquitination, and that it regulates ubiquitination through different sites depending on substrates. However, whether HECTD3 regulates inflammation in liver IRI is unclear.

Tumor necrosis factor receptor-associated factor 3 (TRAF3), the other type of E3s, is a representative member of the TRAF family. As an adaptor molecule, TRAF3 associates the TNF receptor family with numerous signaling pathways related to cell survival and stress responses^17^. However, owing to the postnatal lethality of global TRAF3-deficiency, the functions of TRAF3 are identified until the application of gene conditional knockout^18,19^. Mainly, TRAF3 is involved in inflammation and immune responses by regulating NF-κB, MAPK, and type I interferon (IFN-I) pathways^20,21^. Upon bacterial infection, HECTD3 promotes the polyubiquitination of TRAF3, leading to the promotion of type I IFN production and inflammatory response^11^. However, it is unclear whether HETCD3 and TRAF3 interact directly during bacterial inflammation. In addition, whether their interaction in sterile inflammation is unknown. Although a previous study has revealed that TRAF3 may be involved in the regulation of liver damage and inflammation by activating the NF-κB pathway during liver IRI^18^, the specific action site of TRAF3 and its regulator are still unclear.

Given the effect of HOPE on inflammation in DCD liver transplantation and the interaction between HECTD3 and TRAF3 during bacterial inflammation, we speculated that HOPE may regulate the inflammatory injury induced by ischemia-reperfusion (IR) via HECTD3/TRAF3 pathway in DCD liver transplantation. Herein, in this study, we sought to investigate that the effect of HOPE on liver IRI in a DCD rat model and explore the mechanisms of action of HECTD3 and TRAF3 in its pathogenesis, thereby seeking a new therapeutic target for improving IRI in DCD liver transplantation.

## Results

### HOPE improves liver function and alleviates DCD liver injury in rats

**Fig. 1:**
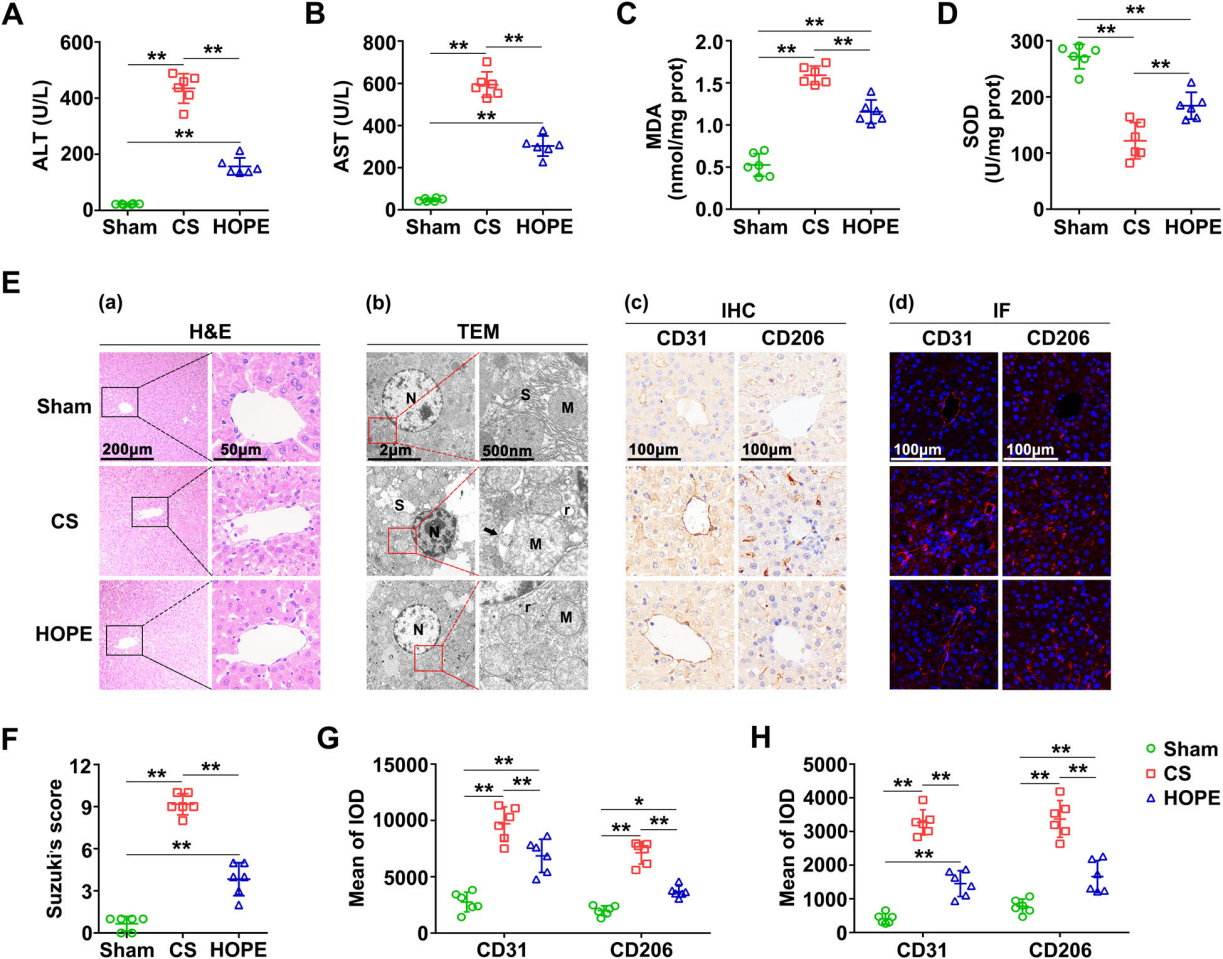
HOPE improves liver function and alleviates DCD liver injury in rats. **A, B** ALT and AST activities in the perfusate. Data are mean ± SD, **P < 0.01 by one-way ANOVA followed by Tukey’s test. n = 6 per group. **C, D** MDA and SOD activities in rat liver tissues. Data are mean ± SD, **P < 0.01 by one-way ANOVA followed by Tukey’s test. n = 6 per group. **E (a)** Histological damage in liver of rats. H&E staining was performed on paraffin-embedded section of rat liver tissues. Scale bar = 200 μm (left panels) and 50 μm (right panels). **E (b)** Changes in the microstructure of hepatocytes. The changes of hepatocellular microstructure were observed using TEM. N, nucleus; M, mitochondria; r, rough endoplasmic reticulum; S, smooth endoplasmic reticulum. The black arrows indicate the lacuna between the inner and outer membranes of mitochondria. Scale bar = 2 μm (left panels) and 500 nm (right panels). **E (c, d)** Activation of LSECs. IHC staining (**c**) and IF staining (**d**) of CD31 and CD206 were performed on paraffin-embedded section of rat liver tissues. Scale bar = 100 μm. **F** Histological scores were analyzed based on Suzuki’s criteria. Data are mean ± SD, **P < 0.01 by one-way ANOVA followed by Tukey’s test. *n* = 6 per group. **G, H** IHC and IF stained fluorescence of CD31 and CD206 were quantified using Image-pro plus 6.0. Data are mean ± SD, *P < 0.05 and **P < 0.01 by two-way ANOVA followed by Tukey’s test. *n* = 6 per group.

To verify the effect of HOPE on liver function in a DCD rat model, we measured alanine aminotransferase (ALT) and aspartate aminotransferase (AST) activities in perfusate. Their activities were significantly increased in the CS group compared with the sham group, whereas these increases were suppressed in the HOPE group (Fig. 1A, B). Moreover, haematoxylin-eosin (H&E) staining of the liver tissues and their histological scores confirmed that HOPE can reduce liver IRI in a DCD rat model (Fig. 1Ea, F).

To explore the effect of HOPE on oxidative stress in liver IRI, we first tested the activities of malondialdehyde (MDA) and superoxide dismutase (SOD) in liver tissues. Compared with the sham group, the CS group showed a distinct increase in MDA activity, and this increase was suppressed in the HOPE group (Fig. 1C). Conversely, that of SOD was higher in the HOPE group than in the CS group (Fig. 1D). Transmission electron microscope (TEM) results showed the deep staining of the nuclear chromatin, clear disappearance of the mitochondrial cristae, and large intermembrane space between the inner and outer mitochondrial membranes; these changes were the most prominent in the CS group (Fig. 1Eb). In addition, the swelling of the smooth endoplasmic reticulum (SER) and increased rough endoplasmic reticulum (rER) were more pronounced in the CS group than in the HOPE group (Fig. 1Eb), indicating that HOPE could inhibit oxidative stress in a DCD rat model.

To explore the effect of HOPE on liver sinusoidal endothelial cells (LSECs), we performed immunohistochemistry (IHC) and immunofluorescence (IF) staining of CD31 and CD206. The staining results showed that the expression levels of CD31 and CD206 were increased in the CS group compared with the sham group, whereas this increase was inhibited in the HOPE group (Fig. 1Ec, Ed, G, H), suggesting that HOPE could improve the LSECs damage-induced by liver IRI.

Collectively, these findings indicate that HOPE could alleviate DCD liver IRI, including the damage to hepatocyte and LSECs.

### HOPE reduces DCD liver inflammation in rats

**Fig. 2:**
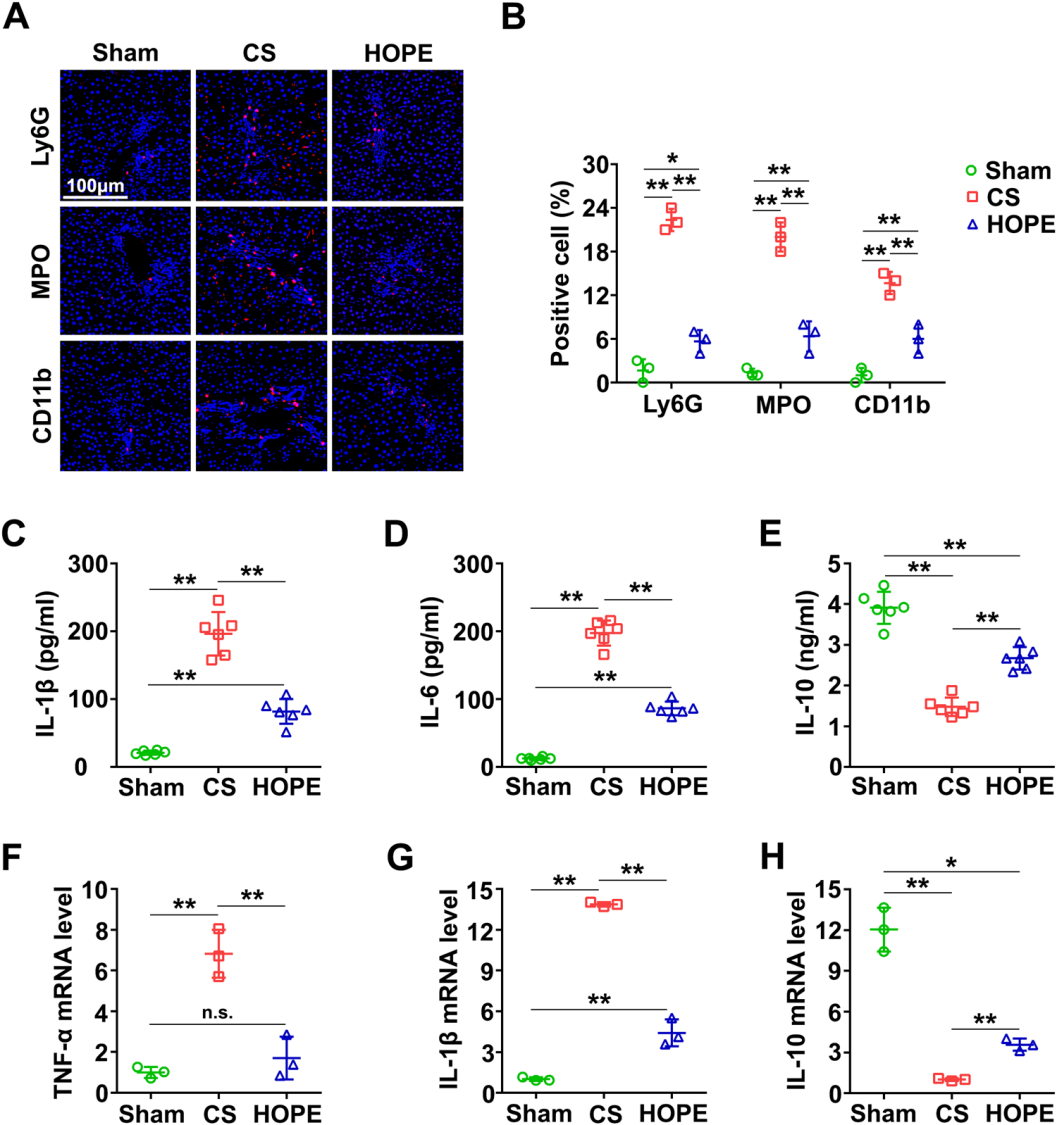
HOPE reduces DCD liver inflammation in rats. **A** IF staining of Ly6G, MPO and CD11b were performed on paraffin-embedded section of rat liver tissues. Scale bar = 100 μm. **B** IF stained fluorescence of Ly6G, MPO and CD11b were quantified using Image-pro plus 6.0. Data are mean ± SD, *P < 0.05 and **P < 0.01 by two-way ANOVA followed by Tukey’s test. *n* = 3 per group. **C**–**E** Liver tissues expression levels of IL-1β, IL-6, and IL-10 were analyzed with the relative ELISA kits. Data are mean ± SD, **P < 0.01 by one-way ANOVA followed by Tukey’s test. *n* = 6 per group. **F**–**H** mRNA expression level in liver of TNF-α, IL-1β, and IL-10 were tested by real-time PCR. Data are mean ± SD, n.s., not significant; *P < 0.05 and **P < 0.01 by one-way ANOVA followed by Tukey’s test. *n* = 3 per group.

To explore the underlying mechanism of the protective effect of HOPE in DCD liver IRI, we conducted IF staining of Ly6G, MPO, and CD11b, which are markers of neutrophil infiltration. Aggregation of neutrophils was significantly higher in the CS group than that in the HOPE group (Fig. 2A, B). Moreover, compared with the CS group, HOPE inhibited the expression of interleukin (IL)-1β and IL-6 (Fig. 2C, D). However, that of IL-10 was higher in the HOPE group than in the CS group (Fig. 2E). The messenger RNA (mRNA) levels of tumor necrosis factor-α (TNF-α) and IL-1β were higher in the CS group than in the HOPE group, whereas that of IL-10 showed the opposite result (Fig. 2F–H). Taken together, these data suggest that HOPE reduces DCD liver IRI in rats may by inhibiting inflammation.

### HOPE inhibits the expression of HECTD3

**Fig. 3:**
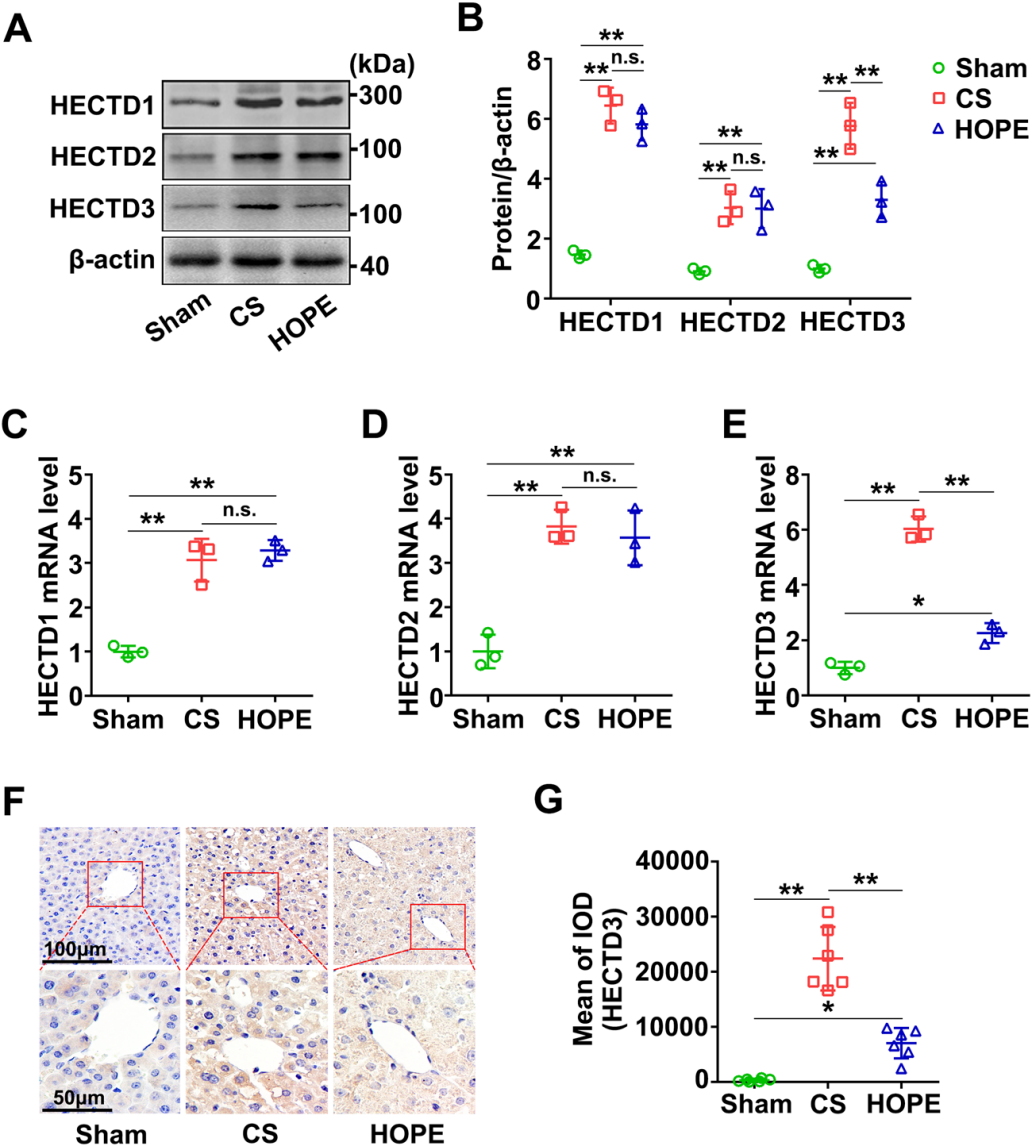
HOPE inhibits the expression of HECTD3 in a DCD rat model. **A, B** HECTD1, HECTD2, and HECTD3 proteins expression in liver were evaluated using western blot (**A**) and quantified using a Gel-Pro Analyzer (**B**). Data are mean ± SD, n.s., not significant; **P < 0.01 by two-way ANOVA followed by Tukey’s test. *n* = 3 per group. **C**–**E** mRNA expression level in liver of HECTD1, HECTD2 and HECTD3 were tested by real-time PCR. Data are mean ± SD, n.s., not significant; *P < 0.05 and **P < 0.01 by one-way ANOVA followed by Tukey’s test. *n* = 3 per group. **F** IHC staining of HECTD3 were performed on paraffin-embedded section of rat liver tissues. Scale bar = 100 μm (upper panels) and 50 μm (down panels). **G** IHC stained fluorescence of HECTD3 was quantified using Image-pro plus 6.0. Data are mean ± SD, *P < 0.05 and **P < 0.01 by one-way ANOVA followed by Tukey’s test. *n* = 6 per group.

Next, we evaluated the expression of HECTD1, HECTD2, and HECTD3 in liver tissues via western blot. The expression levels of HECTD1, HECTD2, and HECTD3 were evidently higher in the CS and HOPE groups than in the sham group, however, those of HECTD1 and HECTD2 were not significantly different between the CS and HOPE groups, except for HECTD3 (Fig. 3A, B). Similarly, their mRNA levels showed this trend (Fig. 3C–E). IHC results further verified that the expression level of HECTD3 in the HOPE group was significantly lower than that in the CS group (Fig. 3F, G). Overall, these suggest that HOPE can inhibit the expression of HECTD3, but not HECTD1 and HECTD2, compared with CS in a DCD rat model.

### HOPE reduces DCD liver injury and inflammation by inhibiting HECTD3 in rats

To further explore whether HOPE reduces liver IRI and inflammation by targeting HECTD3, we reconstructed and in vivo transfected adeno-associated virus 8 (AAV8) to regulate HECTD3, including the HECTD3-overexpression (ov-HECTD3), AAV8 to inhibit its expression (sh-HECTD3) and the corresponding AAV8 empty virus (AAV8 NC). We found that the fluorescence intensity of GFP was the highest on day 12 after transfection (Fig. S1A, B). Moreover, the transfection of AAV8 NC had no significant effect on ATL and AST activities (Fig. S1C, D). Furthermore, compared with the sham and AAV8 NC pre-treatment groups, the ov-HECTD3 AAV8 transfected group had significantly increased HECTD3 expression, whereas the sh-HECTD3 AAV8 transfection group inhibited HECTD3 expression (Fig. S1E). Therefore, in subsequent experiments, we determined the transfection time to be 12 days.

**Fig. 4:**
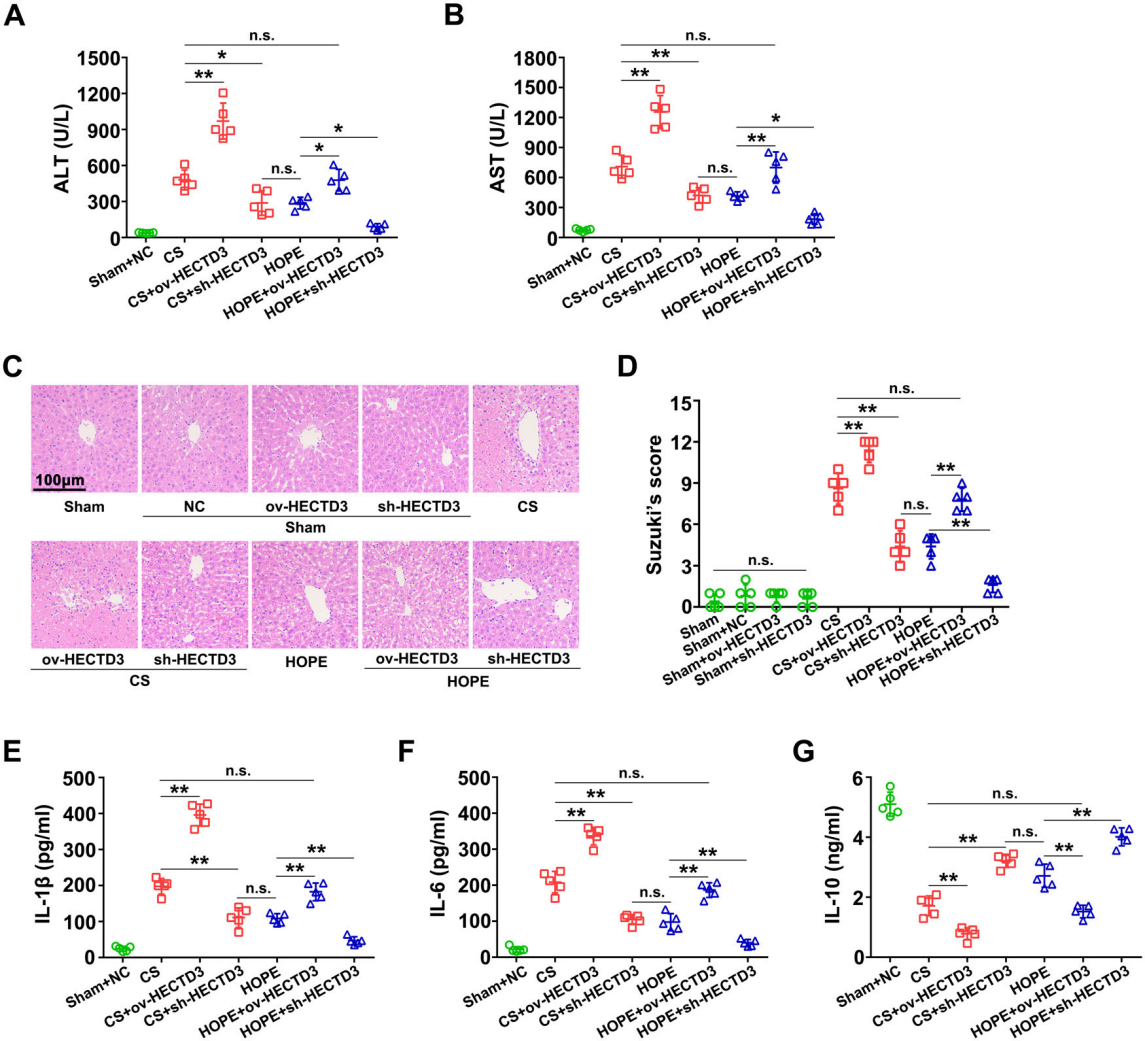
HOPE reduces DCD liver IRI and inflammation by inhibiting HECTD3 in rats. Rats were infected with ov-HECTD3 or sh-HECTD3 AAV8. The rats in the sham group were infected with or without AAV8 NC served as the negative control. After the specimen collection, the following analysises were performed. **A**, **B** ALT and AST activities in perfusate. Data are mean ± SD, n.s., not significant; *P < 0.05 and **P < 0.01 by one-way ANOVA followed by Tukey’s test. *n* = 5 per group. **C** Histological damage in liver of rats. H&E staining was performed on paraffin-embedded section of rat liver tissues. Scale bar = 100 μm. **D** Histological scores were analyzed according to Suzuki’s criteria. Data are mean ± SD, n.s., not significant; **P < 0.01 by one-way ANOVA followed by Tukey’s test. *n* = 5 per group. **E**–**G** Liver tissues expression levels of IL-1β, IL-6, and IL-10 were analyzed with the relative ELISA kits. Data are mean ± SD, n.s., not significant; **P < 0.01 by one-way ANOVA followed by Tukey’s test. *n* = 5 per group.

To explore the role of HECTD3 in HOPE in improving DCD liver IRI, we examined the ALT and AST activities in the perfusate after transfection. ALT and AST activities significantly increased after pre-treatment with ov-HECTD3 AAV8, whereas that with sh-HECTD3 AAV8 had the opposite effect (Fig. 4A, B). However, compared with the CS group, the HOPE group pretreated with ov-HECTD3 AAV8 reversed the effects of HOPE (Fig. 4A, B). Consistently, compared with the HOPE group, ALT and AST activities were not significantly different in the CS group pretreated with sh-HECTD3 AAV8 (Fig. 4A, B). Meanwhile, we confirmed that ov-HECTD3 and sh-HECTD3 AAV8 had no effect on ALT and AST activities (Fig. S1F, G). In line with this, H&E staining of the liver tissues and the histological scores confirmed that pre-treatment with ov-HECTD3 AAV8 aggravated liver damage, whereas that with sh-HECTD3 AAV8 visibly alleviated liver damage (Fig. 4C, D).

Subsequently, we detected the levels of IL-1β, IL-6, and IL-10 in liver tissues after transfection. Enzyme-linked immunosorbent assays (ELISA) results suggested that pre-treatment with ov-HECTD3 AAV8 promoted the expression of IL-1β and IL-6 but inhibited that of IL-10; however, pre-treatment with sh-HECTD3 AAV8 had the opposite effects (Fig. 4E–G). We further verified that ov-HECTD3 and sh-HECTD3 AAV8 had no effect on the expression of IL-1β, IL-6, and IL-10 (Fig. S1H–J). Therefore, our data showed that HOPE reduced liver IRI and inflammation by targeting HECTD3 in a DCD rat model.

### HOPE regulates TRAF3 polyubiquitination by targeting HECTD3

**Fig. 5:**
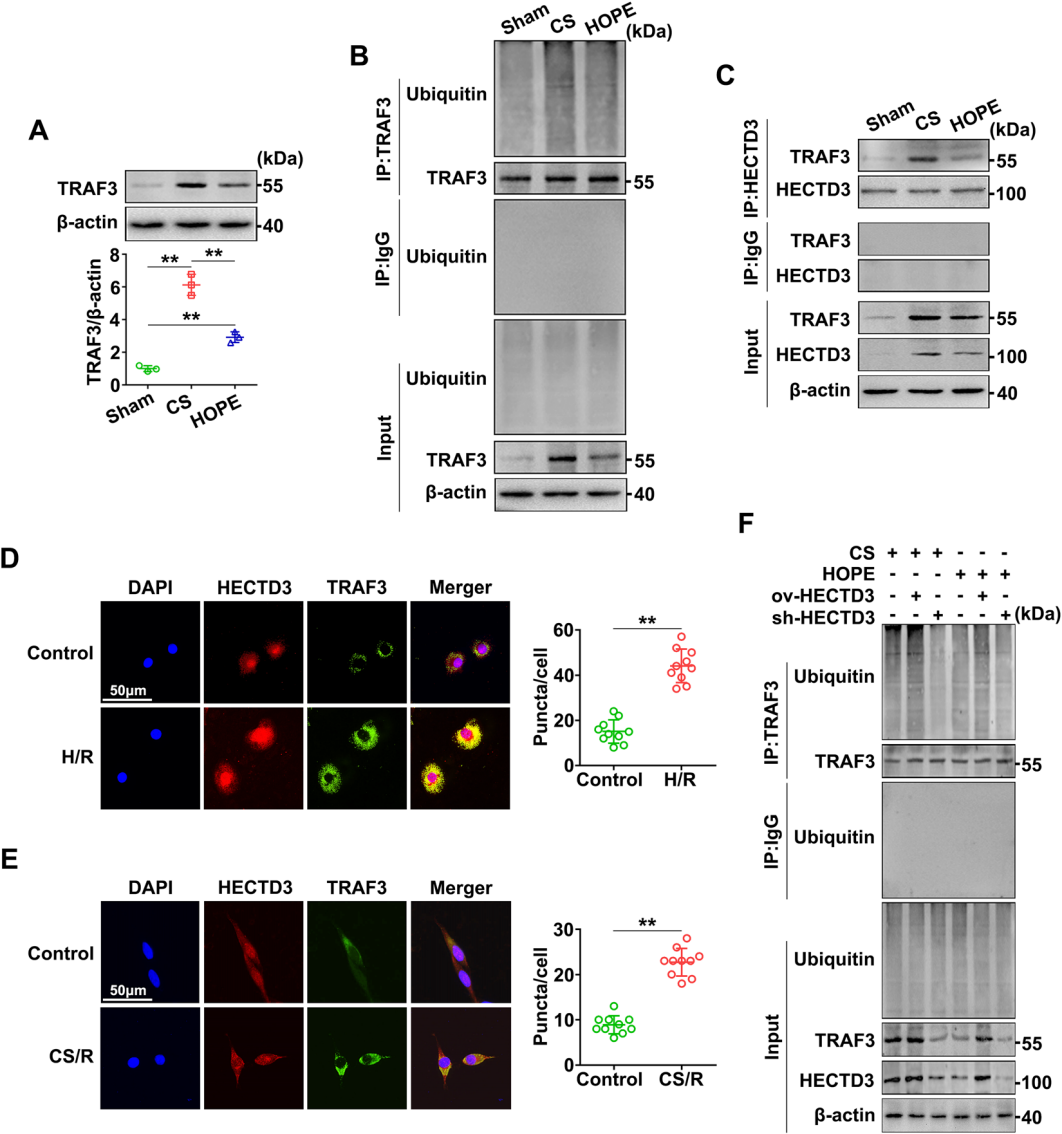
HOPE inhibits the ubiquitination of TRAF3 by targeting HECTD3. **A** TRAF3 protein expression in liver was evaluated using western blot and quantified using a Gel-Pro Analyzer. Data are mean ± SD, **P < 0.01 by one-way ANOVA followed by Tukey’s test. *n* = 3 per group. **B**, **C** Ubiquitination analysis of TRAF3 and Co-IP analysis of the interaction between HECTD3 and TRAF3. Liver tissues protein extracts were IP with primary antibody for TRAF3 (**B**) or HECTD3 (**C**). The immunoprecipitates were blotted with the relative antibodies. **D**, **E** Co-localization of HECTD3 and TRAF3 in hepatocyte and LSECs. IF staining of HECTD3 and TRAF3 were examined in BRL-3A cells with or without H/R and examined in LSECs with or without CS/R. DAPI was used to counter stain nuclei. The stained fluorescence was observed using a fluorescence microscope and quantified using Image-pro plus 6.0. Scale bar = 50 μm. Data are mean ± SD, **P < 0.01 by Student’s two-tailed unpaired *t*-test. *n* = 10 per group. **F** Ubiquitination analysis for the regulation of endogenous TRAF3 by HECTD3. Partial groups of rats were infected with ov-HECTD3 or sh-HECTD3 AAV8. Liver tissues protein extracts were immunoprecipitated with primary antibody for TRAF3. The immunoprecipitates were blotted with the relative antibodies.

To explore the underlying mechanism of HECTD3 reduction in liver IRI and inflammation, we next analyzed the expression and activation of TRAF3. Western blot analysis indicated that the expression of TRAF3 was distinctly increased in the CS group, and this effect was suppressed in the HOPE group (Fig. 5A). Furthermore, we found that TRAF3 polyubiquitination was remarkably increased in the CS group; however, this effect was inhibited in the HOPE group (Fig. 5B). These suggest that HOPE suppressed the polyubiquitination of TRAF3 in a DCD rat model.

To confirm whether HECTD3 and TRAF3 interacts in the DCD rat model, we performed co-immunoprecipitation (Co-IP) analysis. Compared with the CS group, HOPE partly inhibited the interaction between HECTD3 and TRAF3 (Fig. 5C). To further verify this interaction, we established a hypoxia/reoxygenation (H/R) model of BRL-3A cells and a cold storage/reoxygenation (CS/R) model of LSECs to simulate in vivo warm IR and cold IR, respectively. Compared with the control group, H/R and CS/R visibly promoted the co-localization of HECTD3 and TRAF3 in BRL-3A cells and LSECs, respectively (Fig. 5D, E). Therefore, our in vivo and in vitro experiments confirmed the inevitable interaction between HECTD3 and TRAF3.

To further explore whether HECTD3 regulates the polyubiquitination of TRAF3, we conducted the ubiquitination analysis after rats were transfected with ov-HECTD3 and sh-HECTD3 AAV8. We found that pre-treatment with ov-HECTD3 AAV8 promoted the TRAF3 polyubiquitination, whereas pre-treatment with sh-HECTD3 AAV8 visibly inhibited this effect (Fig. 5F). Thus, these data suggest that HOPE inhibits the polyubiquitination of TRAF3 by targeting HECTD3.

### Interaction between HECTD3 and TRAF3

**Fig. 6:**
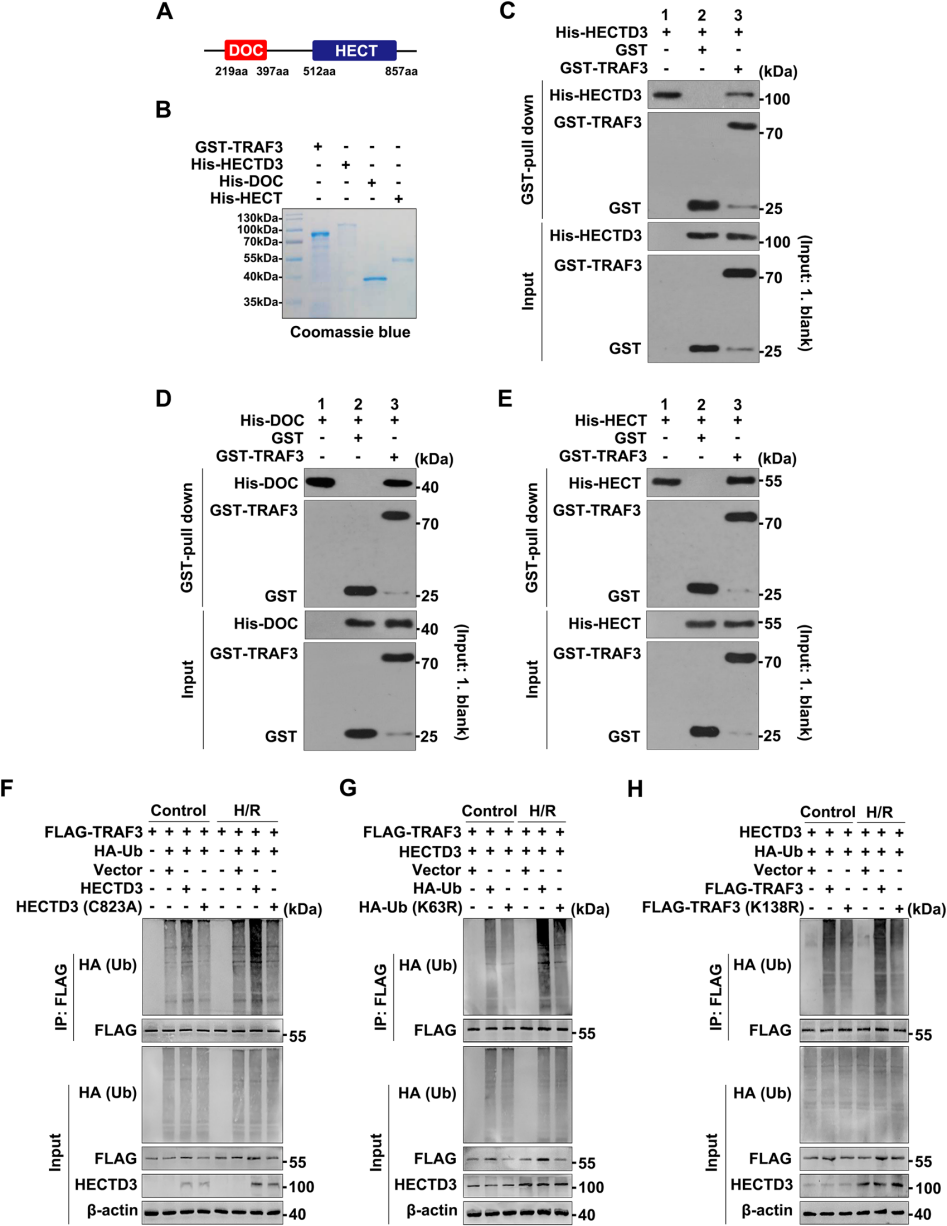
Interaction between HECTD3 and TRAF3. **A** Schematic diagram of the domain architecture of HECTD3. The DOC domain is located at the amino acid (aa) sequence 219 to 397, whereas the HECT domain is located at the amino acid (aa) sequence 512 to 857. **B** Coomassie-stained SDS-PAGE of purified TRAF3 and recombinant HECTD3 constructs: GST-tagged TRAF3 and His-tagged full-length HECTD3, His-tagged isolated DOC and HECT domains. **C**–**E** GST pull-down assay with purified GST-TRAF3 and recombinant His-tagged full-length HECTD3, His-tagged isolated DOC and HECT domains. Purified GST-tagged TRAF3 was incubated with His-tagged full-length HECTD3 (**C**), the isolated DOC (**D**) and HECT domains (**E**). The interaction between TRAF3 and HECTD3, the DOC and HECT domains were visualized using immunoblots. **F**–**H** Ubiquitination analysis of TRAF3 and the interaction sites between HECTD3, Ub and TRAF3. BRL-3A cells transfected with mutants HECTD3 (C823A), HA-tagged Ub (K63R) and FLAG-tagged TRAF3 (K138R) plasmids or the corresponding WT and vector plasmids. Cellular protein extracts in BRL-3A cells with or without H/R were immunoprecipitated with primary antibody for FLAG. The immunoprecipitates were blotted with the relative antibodies.

To clarify the interaction between HECTD3 and TRAF3 in liver IRI, we first showed the schematic diagram of the domain architecture of HECTD3 (Fig. 6A). Subsequently, glutathione S-transferase (GST) pull-down assays demonstrated that the GST-TRAF3 protein, but not GST, was pulled down by exogenous His-HECTD3, His-DOC, His-HECT (Fig. 6B–E), indicating that both full-length HECTD3 and isolated DOC and HECT domains directly interacted with TRAF3.

To pinpoint the binding site between HECTD3 and TRAF3 and identify the type of TRAF3 polyubiquitination regulated by HECTD3 in H/R, we conducted the following experiments. First, to verify the catalytic cysteine (C832) in the HECT domain essential for TRAF3 polyubiquitination, we constructed a HECTD3 mutant HECTD3 (C823A). Subsequent ubiquitination analysis showed that the polyubiquitination level of TRAF3 in the presence of HECTD3 (C823A) was lower than that of HECTD3 in the control and H/R groups, and that the overall level of in the H/R group was higher than that of in the control group (Fig. 6F). Second, to identify the type of TRAF3 polyubiquitination regulated by HECTD3 in H/R, we constructed a HA-ubiquitin (Ub) mutant HA-Ub (K63R). Compared with the group transfected with HA-Ub, that with HA-Ub (K63R) inhibited the polyubiquitination of TRAF3, and the overall level of the H/R group was higher than that of the control group (Fig. 6G). Finally, to identify the lysine (Lys)138 residue is essential for HECTD3-mediated K63-linked polyubiquitination of TRAF3, we constructed a TRAF3 mutant TRAF3 (K138R). Evidently, TRAF3 polyubiquitination was diminished in K138R in both the control and H/R groups (Fig. 6H). Overall, these data suggested that the C832 of the HECT domain promotes the K63-linked polyubiquitination of TRAF3 at Lys138 in BRL-3A cells under H/R conditions.

### TRAF3 Lys138 is essential for regulating H/R-induced injury in BRL-3A cells

**Fig. 7:**
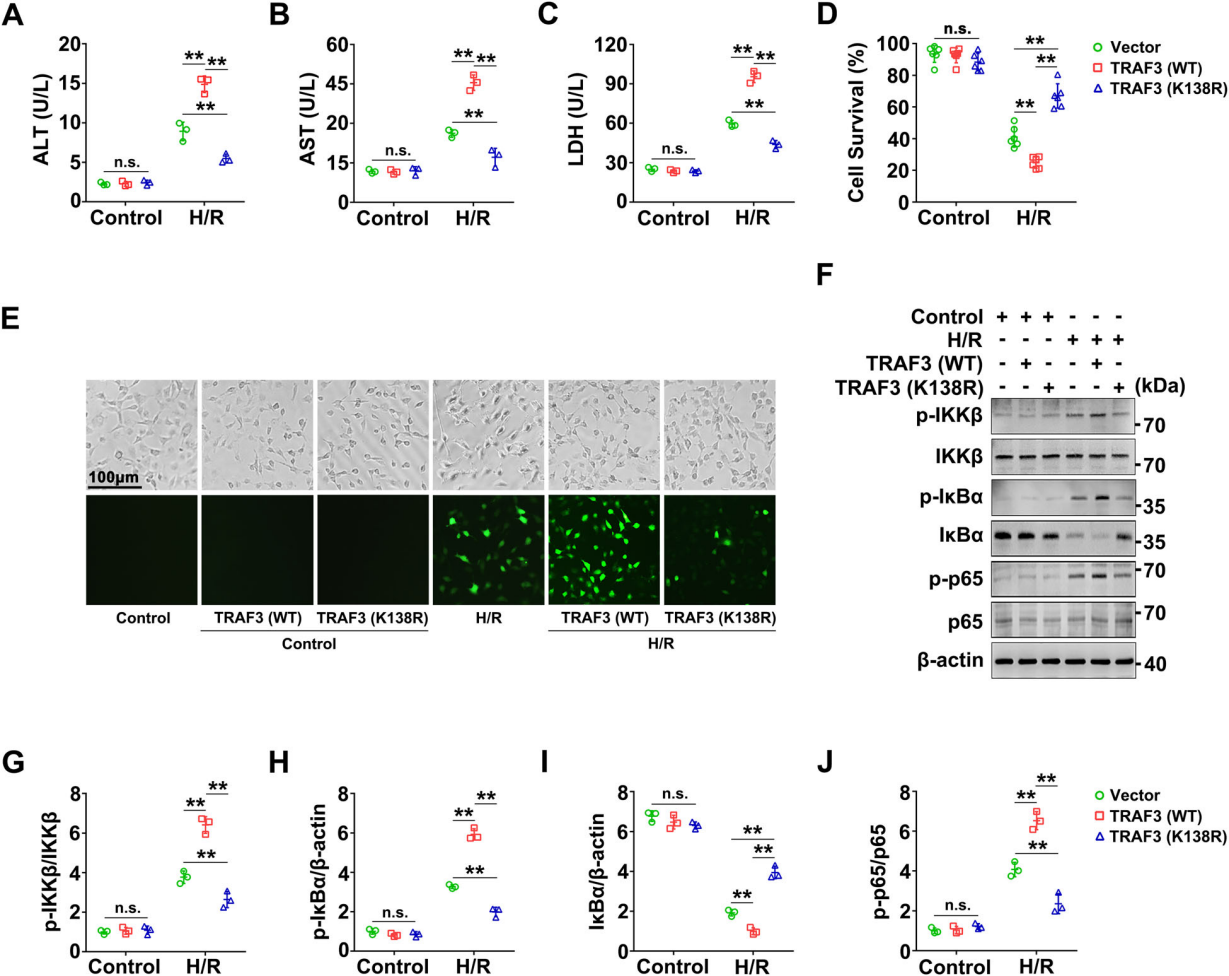
TRAF3 Lys138 mutants reduce the injury of BRL-3A cells with H/R. BRL-3A cells transfected with TRAF3 Lys138 mutants (K138R) plasmids or the corresponding WT and vector plasmids as indicated, and the cells were cultured under normal or H/R conditions. The following analysises were performed. **A**–**C** ALT, AST, and LDH activities in the culture medium. Pretreated BRL-3A cells were incubated under normal or H/R conditions. Data are mean ± SD, n.s., not significant; **P < 0.01 by two-way ANOVA followed by Tukey’s test. *n* = 3 per group. **D** CCK-8 assay was used to examine cell survival. Data are mean ± SD, n.s., not significant; **P < 0.01 by two-way ANOVA followed by Tukey’s test. *n* = 6 per group. **E** ROS in BRL-3A cells. ROS staining was performed to evaluate the level of ROS in BRL-3A cells, and the stained fluorescence was observed using a fluorescence microscope. The cellular morphology and structure of BRL-3A cells by bright image investigation. **F**–**J** Proteins levels of p-IKKβ, IKKβ, p-IκBα, IκBα, p-p65, p65 in BRL-3A cells were evaluated using western blot (**F**) and quantified using a Gel-Pro Analyzer (**G**–**J**). Data are mean ± SD, n.s., not significant; **P < 0.01 by two-way ANOVA followed by Tukey’s test. *n* = 3 per group.

To explore the role of TRAF3 Lys138 in H/R, we transfected BRL-3A cells with TRAF3 (WT) and TRAF3 (K138R) plasmids. Through the assays of ALT, AST and lactate dehydrogenase (LDH) activities in the culture medium, we found that the ALT, AST, and LDH activities in the presence of TRAF3 (K138R) were lower than those of TRAF3 (WT) in the H/R groups, whereas there was no significant difference in the control groups (Fig. 7A–C). These findings indicate that TRAF3 Lys138 is essential for regulating the liver function injury induced by H/R in BRL-3A cells. Moreover, cell counting kit-8 (CCK-8) analysis showed that the cell survival rate significantly increased in the presence of TRAF3 (K138R) than that of TRAF3 (WT) in the H/R groups (Fig. 7D), indicating that TRAF3 Lys138 is crucial for cell survival in the H/R of BRL-3A cells.

To elucidate the mechanism by which the TRAF3 Lys138 mutant attenuated cell death in BRL-3A cells under H/R conditions, we evaluated the accumulation of reactive oxygen species (ROS) in BRL-3A cells. There was a significantly increase in ROS accumulation in the group transfected with TRAF3 (WT) than in that with TRAF3 (K138R) under H/R conditions (Fig. 7E), suggesting that TRAF3 Lys138 mutants prominently inhibited oxidative stress in H/R. In addition, BRL-3A cells in H/R transfected with TRAF3 (K138R) inhibited the phosphorylation of IKKβ, IκBα, and p65 at sites Tyr199, Ser32/Ser36, and Ser536, respectively (Fig. 7F–J), which are vital proteins in the NF-κB signaling pathway. Thus, the TRAF3 Lys138 mutants inhibited the activation of the NF-κB signaling pathway in BRL-3A cells subjected to H/R. In summary, the TRAF3 Lys138 mutant is essential for suppressing oxidative stress and inflammation induced by H/R in BRL-3A cells.

### Human liver IR promotes the expression of HECTD3 and TRAF3

**Fig. 8:**
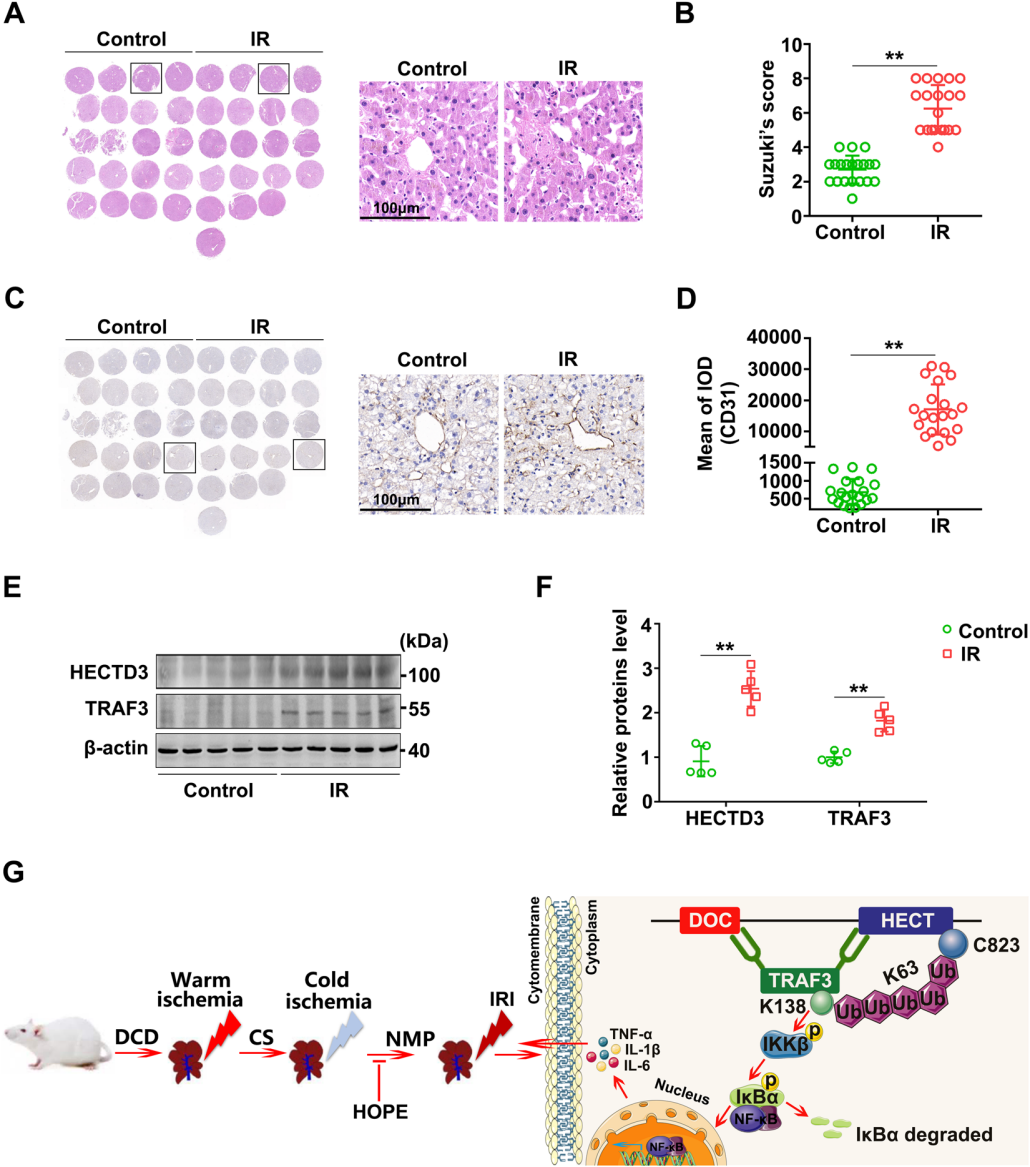
Human liver IRI promotes the expression of HECTD3 and TRAF3. **A, B** Histological damage in human liver. Overview of a TMA section stained with H&E. Histological change was observed using a microscope (**A**). The right panel is a representative enlarged image. Scale bar = 100 μm. Histological scores were analyzed depending on Suzuki’s criteria (**B**). Data are mean ± SD, **P < 0.01 by Student’s two-tailed unpaired *t*-test. *n* = 20 per group. **C**, **D** Activation of LSECs in human liver. Overview of a TMA section stained with IHC. The right panel is a representative enlarged image. Scale bar = 100 μm. IHC stained fluorescence of CD31 was quantified using Image-pro plus 6.0. Data are mean ± SD, **P < 0.01 by Student’s two-tailed unpaired *t*-test. *n* = 20 per group. **E**, **F** HECTD3 and TRAF3 proteins expression in human liver were evaluated using western blot (**E**) and quantified using a Gel-Pro Analyzer (**F**). Data are mean ± SD, n.s., not significant; **P < 0.01 by two-way ANOVA followed by Tukey’s test. *n* = 5 per group. **G** Mechanism scheme. Warm ischemia, cold ischemia, and subsequent reperfusion phases in a DCD rat model cause the DOC and HECT domains of HECTD3 directly binding to TRAF3. Followed by the C823 of the HECT domains promotes the K63-linked polyubiquitination of TRAF3 at Lys138 to trigger the activation of NF-κB signaling pathway, leading to the release of inflammatory factors to result in liver IRI. However, HOPE effectively improved the entire pathological process.

To verify the changes in HECTD3 and TRAF3 levels in human livers suffering from IR, we collected five pairs of DCD liver transplantation specimens. The donor liver underwent warm and cold ischemia for almost a period of time and a one-hour reperfusion (Table 1). Through H&E staining of tissue microarray (TMA) and histological scores, we found that IR caused varying degrees of pathological damage to human liver tissues (Fig. 8A, B). Moreover, the expression of CD31 visibly increased in the IR group compared with the control group (Fig. 8C, D), suggesting that the LSECs in human liver tissues were damaged by IR. Further, western blot results showed that the expression levels of HECTD3 and TRAF3 in the IR group were significantly higher than those in the control group (Fig. 8E, F), suggesting that human liver IR during DCD liver transplantation promotes the expression of HECTD3 and TRAF3.

**Table 1:** Donor and recipient information for liver transplantation.

Donor						Recipient		
Number	Age (years)	Gender	Total ischemic time (h)	Warm ischemia time (min)	Cold ischemia time (h)	Age (years)	Gender	Reperfusion time (h)
1	57	Male	7.4	14	7.2	27	Male	1
2	63	Male	8.2	12	8.0	48	Male	1
3	71	Male	12.0	12	11.8	40	Male	1
4	16	Male	8.0	15	7.7	49	Male	1
5	67	Male	9.7	16	9.4	50	Male	1

## Discussion

IRI during DCD liver transplantation can lead to up to 10% of early transplant failures and increase graft rejections^22^. We and other researchers have previously demonstrated that HOPE has a significant improved liver IRI after transplantation by regulating inflammation^1,8,10^; however, the underlying protective mechanism is unclear. Our results of this study demonstrated that HOPE reduces inflammation by regulating the HECTD3/TRAF3 signaling pathway to improve DCD liver IRI.

Studies on rat models of DCD liver transplantation showed that warm ischemia can cause significant damage to hepatocytes, especially mitochondria, and this damage is aggravated with prolonged ischemia^23^. In this study, we found that these injuries were alleviated after HOPE treatment, indicating that HOPE significantly improved hepatocyte injury in a DCD rat model. In contrast, the damage caused by cold ischemia mainly targets LSECs^24,25^. Studies have shown that CS can cause morphological changes in LSECs, including the retraction and detachment of cell bodies. Subsequent reperfusion can aggravate this damage and result in the almost complete deprivation of the LSECs lining^25^. Similarly, this study confirmed that CS can significantly increase the expression of CD31 and CD206, important markers of LSECs damage^26–28^. Surprisingly, the damage was visibly suppressed after HOPE treatment, suggesting that HOPE can also improve LSECs damage in a DCD rat model.

In previous research, we have verified that HOPE can reduce liver IRI by inhibiting oxidative stress and inflammation^8,10^. In addition, a study on fatty liver grafts showed that HOPE inhibits nuclear damage-associated molecular patterns (DAMPs) trigger, and the activation of toll-like receptors and stellate cells, thereby decreasing cellular inflammation^1^. However, the underlying mechanism of HOPE in improving liver IRI has not been thoroughly explored in this study. In the present study, to better simulate the clinical liver transplantation setting, we extended the duration of CS and immediately performed HOPE treatment in rat livers. Surprisingly, HOPE treatment consistently reduced the liver inflammation and injury induced by long-term CS and the subsequent reperfusion, thereby confirming that HOPE can improve DCD liver IRI in rats by targeting inflammation. Furthermore, we found that HECTD3 and TRAF3, two different types of E3s, are indispensable in the regulation of HOPE for DCD liver IRI in rats.

Presently, little is known about the function of HECTD3 (ref. ^29^). According to current research, HECTD3 plays an important role in regulating tumors, as well as inflammation and immune-related diseases, by interacting with other proteins^13,16,29–31^. In a study on EAE, HECTD3 promoted the nondegradative polyubiquitination of Stat3 and Malt1 to increase the upregulation of RORγt and the activation of NF-κB, thus aggravating neuroinflammation^16^. This indicates that HECTD3 plays a proinflammatory role in EAE. Similarly, here, we found that the overexpression of HECTD3 promoted DCD liver injury and inflammation in rats, whereas its inhibition had the opposite effect. Interestingly, the expression of HECTD3, but not of HECTD1 and HECTD2, was significantly different between the CS and HOPE groups. Structurally, both HECTD1 and HECTD2 do not contain a unique DOC domain, which is responsible for binding to the substrate to play more biological functions^12,14^. Functionally, HECTD1 is responsible for neural tube closure during embryonic development^32^, whereas HECTD2 may be mainly related to HPV-induced cervical cancers and Angelman syndrome^33^. Therefore, HOPE targets HECTD3, but not HECTD1 and HECTD2, to regulate inflammation, thereby improving the liver IRI-induced by DCD in rats.

Significant progress has been made in studying the role of E3s in disease progression during bacterial and viral infections. Once infected with DNA or RNA viruses and various bacteria, some RING-type E3s play positive or negative roles in the pathological processes by regulating different types of polyubiquitination^11,34,35^. However, the role of HECT-type E3s remains unclear. Recent studies have shown that HECTD3 promotes the polyubiquitination of TRAF3 to modulate type I interferon induction upon infection with intracellular bacteria^11^. However, the study did not examine whether HECTD3 directly interacts with TRAF3. Moreover, the interaction between HECTD3 and TRAF3 remains undocumented in sterile inflammation, which is a major clinical concern during liver IRI, can be triggered by various danger molecules, including DAMPs, pattern recognition receptors (PRRs), and high mobility group box 1 (HMGB1) (ref. ^7,36^). Here, we found that the HECT domain, which is mainly responsible for the catalytic function of HECTD3, can also directly bind to TRAF3, in addition to the DOC domain. However, another study showed that only the DOC domain directly combines with CRAF and HSP90 (ref. ^30^). Hence, the function of the HECT domain of HECTD3 may vary depending on the specific substrate. Further, we verified that the C823 of the HECT domain promoted the K63-linked polyubiquitination of TRAF3 to regulate inflammation in BRL-3A cells under H/R conditions. Therefore, the interaction between HECTD3 and TRAF3 plays an important role in regulating inflammation in both bacterial and sterile conditions.

As a representative member of RING-type E3s, TRAF3 plays an indispensable role in regulating immune and inflammation-related diseases. Ubiquitin modification is the main form of TRAF3 activation. The polyubiquitin chains link to lysine residues mainly including K6, K11, K27, K29, K33, K48, and K63 (ref. ^37^), depending on the specific context, thereby playing different roles. For instance, K48-linked ubiquitination is responsible for substrate degradation, whereas K63-linked ubiquitination is responsible for downstream signaling activation and protein trafficking^38^. Specifically, cIAPs, an E3 ubiquitin ligase, promotes K48-linked polyubiquitination of TRAF3 for proteasomal degradation, which is a novel mechanism for paricalcitol, a vitamin D receptor agonist, to inhibit inflammation in renal diseases^39^. Moreover, Mint3, a unique member of the Mint protein family, promotes the K63-linked polyubiquitination of TRAF3 to enhance IRF3 activation and IFN-β production induced by TLR3/4 and RIG-I (ref. ^40^). Overtly, different regulators of TRAF3 have different effects through various lysine residues linking polyubiquitination of TRAF3 under different conditions. In this study, we showed that increased HECTD3 promoted the K63-linked polyubiquitination of TRAF3 at Lys138 in BRL-3A cells subjected to H/R. Moreover, this increased polyubiquitination aggravated liver function and reduced the survival of BRL-3A cells under H/R conditions by regulating oxidative stress and the NF-κB signaling pathway. These data suggest that targeting HECTD3 regulated K63-linked polyubiquitination of TRAF3 at Lys138 may be an important mechanism for regulating the injury of BRL-3A cells during H/R. Finally, the increased expression of HECTD3 and TRAF3 was confirmed in human liver specimens subjected to IRI in DCD liver transplantation. Unfortunately, we could not perform HOPE treatment on theses human specimens because the HOPE system applied in the clinical setting is still under investigation.

In conclusion, we showed that the HECTD3/TRAF3 signaling pathway played an essential role in HOPE in improving liver IRI by inhibiting inflammation in a DCD rat model. Particularly, upon DCD liver IR insult, the DOC and HECT domains of HECTD3 directly bind to TRAF3, and the C832 of the HECT domain promotes the K63-linked polyubiquitination of TRAF3 at Lys138 to trigger oxidative stress and inflammatory response, thereby leading to serious liver IRI. However, HOPE effectively improved the entire pathological process. Therefore, HOPE targeting the HECTD3/TRAF3 signaling pathway represents a promising therapeutic target for liver IRI in DCD liver transplantation.

## Materials and methods

### Establishment of DCD rat model

Adult male Sprague-Dawley (SD) rats (8- to 10-week-old; weight: 250–300 g) were obtained from Wuhan Wan Qian Jia Xing Bio-Technology Co., Ltd. (Hubei, China). The rats were provided with suitable food and water and housed in an environment with controlled humidity, temperature, and light (12:12 light/dark cycle).

The DCD rat model was generated as previously described^5,8^. Briefly, the rats were randomly divided into different groups and the investigator was not blinded to group allocation during the experiment. The rats were administered with anesthesia via intraperitoneal injection of pentobarbital sodium salt (30 mg/kg body mass) before midline laparotomy. Subsequently, cardiac death was caused by hypoxia after diaphragm incision without prior heparinization or portal clamping^41^. The in situ warm ischemia phase starts from cardiac arrest. During this phase, the liver temperature was maintained at 29 ± 1.45 °C. After 30 min, to flush out the blood in the livers, 50 mL of 4 °C histidine-tryptophan-ketoglutarate (HTK) solution (Dr. Franz Koehler Chemie GmbH, Bensheim, Germany) was used to perfuse the livers in situ through the abdominal aorta. After the perfusion, the portal vein and superior hepatic caval vein were cannulated, and the infrahepatic vena caval and right adrenal vein were ligated. Finally, the liver was resected.

Subsequently, the isolated livers were subjected to 23 h of CS, and some samples were treated using HOPE for 1 h, whereas the others were not. Then, all livers were subjected to 1 h of normothermic perfusion (NMP). In the sham group, the isolated healthy livers were only subjected to 1 h of NMP without warm ischemia and CS. The HOPE and isolated perfusion rat liver model (IPRL) systems were described in detail in a previous study^8^.

The study protocol was approved by the Ethical Committee of Wuhan University, and all animal experiments were carried out in accordance to the Experimental Animal Management Ordinance (National Science and Technology Committee of China) and the Guide for the Care and Use of Laboratory Animals (National Institutes of Health, Bethesda, MD, USA).

### AAV8 construction and in vivo transfection

Recombinant AAV8 for HECTD3-overexpression (ov-HECTD3), AAV8 to inhibit its expression (sh-HECTD3), and the homologous AAV8 empty virus (AAV8 NC) were purchased from Genechem Biotech Inc. (Shanghai, China). AAV8 was administered via tail-vein injection at a dose of 1.0 × 10^11^ viral genome (v.g.) per rat (1 mL total volume).

### Cell culture and H/R or CS/R model

BRL-3A cells and LSECs (BNBIO., Beijing, China) were cultured in a humidified incubator (Thermo, Marietta, GA, USA) maintained at 37 °C and 5% CO_2_ in high-glucose Dulbecco’s Modified Eagle Medium (DMEM) supplemented with 10% heat-inactivated fetal bovine serum, 1% 100 U/mL penicillin G, and 100 μg/mL streptomycin. To generate the in vitro H/R model, BRL-3A cells were incubated for 12 h in a microaerophilic system (Heal Force, Shanghai, China) containing 5% CO_2_, 1% O_2_, and 94% N_2_ gas. Then, the cells were cultured for 6 h under normoxic conditions to allow reoxygenation. Meanwhile, to generate the in vitro CS/R model, LSECs were cultured for 24 h at 4 °C in cold Celsior or University of Wisconsin (UWS) solutions instead of the normal medium. Subsequently, they were washed twice with cold phosphate-buffered saline (PBS) and cultured for 2 h under normoxic conditions to mimick reperfusion^24^.

### Plasmid construction and in vitro transfection

The TRAF3 (WT), HECTD3 (WT) plasmid, FLAG-tagged TRAF3 plasmid, HA-tagged Ub plasmid, HECTD3 (C823A) plasmid (whose cysteine residue at position 823 was replaced by alanine), HA-tagged Ub (K63R) plasmid (whose lysine residue at position 63 was replaced by arginine), FLAG-tagged TRAF3 (K138R) plasmid (whose lysine residue at position 138 was replaced by arginine), and the empty vector plasmid were synthesized by Genechem Biotech Inc. (Shanghai, China). The BRL-3A cells were transfected with the relevant plasmid or the empty vector plasmid using Lipofectamine 3000 according to the manufacturer’s instructions.

### Biochemical analysis

Blood was collected from the postcava and centrifuged at 3500 rpm for 10 min. The perfusate and liver tissues were collected after normothermic reperfusion, and the serum, perfusate, and liver tissues were stored at −80 °C. The cell culture medium was collected after normal culture or modeling. ALT and AST levels in the rat serum and perfusate were measured using automatic analysis in the Zhongnan Hospital of Wuhan University. MDA and SOD activities in the rat liver were measured using commercial kits (Jiancheng, Nanjing, China) according to the manufacturer’s protocol. The activities of the AST, ALT, and LDH in the cell culture medium were measured using an automatic biochemical analyzer (Rayto, Shenzhen, China).

### H&E staining

Liver tissues were harvested, fixed in 4% paraformaldehyde and embedded in paraffin as previously described^42^. All paraffin sections were stained with H&E for histological observation and the graded blindly according to Suzuki’s criteria^43^. Histological changes were graded from 0 to 4 depended on the severity of cellular vacuolization, hepatic sinusoid congestion, and hepatocyte necrosis.

### TEM assay

Fresh liver tissue fragments (1–2 mm^3^) were immediately fixed in 2.5% glutaraldehyde overnight at 4 °C, washed with 0.1 M PB three times for 30 min each, fixed with 2% osmium tetroxide in 0.1 M PB for 2 h, and dehydrated in graded ethanol solutions (30, 50, 70, 80, 85, 90, 95, and 100%) for 15 min at each concentration. Then, they were infiltrated with propylene oxide (PO) twice for 20 min each and placed into a 70:30 mixture of PO and resin for 1 h. PO was volatilized by keeping the lid of the tube open overnight. The samples were transferred to fresh 100% resin, polymerize at 60 °C for 48 h, cut into ultra-thin sections (70–90 mm), stained with 2% uranyl acetate at room temperature for 15 min, washed with distilled water, and further stained with lead stain solution at room temperature for 3 min. The grids were observed using a transmission electron microscope (Tecnai G^2^ 20 TWIN; FEI, Hillsboro, USA) at an acceleration voltage of 200 kV.

### IHC and IF staining

For IHC, the liver tissues were fixed in 4% paraformaldehyde, embedded in paraffin, and sectioned (4-μm thickness). They were then dewaxed, hydrated for 30 min at room temperature, and incubated overnight at 4 °C with the following primary antibodies: anti-CD31 (1:1000, Proteintech, Wuhan, China); anti-CD206 (1:10000, Proteintech, Wuhan, China), anti-HECTD3 (1:400, BIOS, Beijing, China). Subsequently, the slides were incubated with a horseradish peroxidase-labeled secondary antibody, and the immunoreactivity was visualized with 3,3-diaminobenzidine tetrahydrochloride (DAB). Tissue sections were counterstained with hematoxylin and visualized using Leica Microsystems at 200 × and 400 × magnification. Image-pro plus 6.0 was used for image analysis (Media CybernetiHOPE, Inc., Rockville, MD, USA).

IF was performed as previously reported^44^. Specifically, the sections or cell slides were incubated with anti-CD31, anti-CD206, anti-TRAF3 (1:100, Proteintech, Wuhan, China), anti-Ly6G (1:100, Novus, Littleton, USA), anti-MPO (1:200, Servicebio, Wuhan, China), anti-CD11b (1:500, Servicebio, Wuhan, China) and anti-HECTD3 (1:100, BIOS, Beijing, China) antibodies. After washing, the slides were incubated with Alexa Fluor 594-conjugated secondary antibodies or Alexa Fluor 488-conjugated secondary antibodies (1:200, Proteintech, Wuhan, China).

### Enzyme-linked immunosorbent assays (ELISA)

ELISA commercial kits (MultiSciences Biotech, Hangzhou, China) were used to measure the IL-1β, IL-6, and IL-10 concentrations in rat liver tissues according to the manufacturer’s instructions. The optical density of each well was determined using a microplate reader (Molecular Devices, CA, USA). The detailed description of the specific protocol has been reported previously^45^.

### Quantitative real-time PCR

Quantitative real-time PCR was performed as detailed described previously^8,10^. Briefly, total RNA from frozen rat liver tissues was extracted with TRIzol reagent (Invitrogen Inc., Grand Island, NY) and reverse-transcribed to cDNA (Thermo Scientific Revert Aid). The expressions of the target genes were detected by SYBR Green quantitative real-time polymerase chain reaction, with β-actin as an internal control. The primer sequences used are listed in Supplementary Table 1.

### Western blot analysis

Western blot was performed as previously reported^44^. Briefly, the membranes were incubated overnight at 4 °C with the following corresponding primary antibodies: anti-HECTD1 (1:200, Santa Cruz Biotechnology, Santa Cruz, CA, USA), anti-HECTD2, anti-HECTD3 (1:800, BIOS, Beijing, China), anti-TRAF3 (1:200, Affinity Biosciences, Shanghai, China), anti-phospho-IKKβ (Tyr199), anti-phospho-IκBα (Ser32/Ser36), anti-phospho-p65 (Ser536) (1:500, Affinity Biosciences, Shanghai, China), anti-IKKβ, anti-IκBα, anti-p65 (1:1000, Proteintech, Wuhan, China), anti-GST tag, anti-His tag, anti-FLAG tag, anti-HA tag (1:5000, Proteintech, Wuhan, China), and anti-β-actin (1:3000, Proteintech, Wuhan, China) antibodies.

### Co-IP and ubiquitination analysis

Total proteins were extracted from liver tissues and BRL-3A cells as previously reported^44^. An equal amount of anti-HECTD3 or anti-TRAF3 or anti-FLAG tag antibody was added to 500 μg of protein and gently shaken at 4 °C overnight. Immunocomplexes were acquired by adding 40 μL of protein A + G agarose beads (Beyotime Institute of Biotechnology, Shanghai, China). The mixtures were then gently shaken at 4 °C for 4 h. The mixture was centrifuged at 1000 × *g* for 5 min at 4 °C, and the supernatant was discarded. The sediment was washed five times using ice-cold PBS. The immunocomplexes were boiled in sodium dodecyl sulfate sample buffer for 5 min to separate them from the beads. Then, the specimens were examined via immunoblotting with anti-HECTD3 and anti-TRAF3 antibodies or anti-FLAG tag antibody according to the manufacturer’s instructions. For ubiquitination analysis, the immunocomplexes were analyzed by immunoblotting with an anti-ubiquitin or anti-HA tag antibody.

### Recombinant HECTD3 and TRAF3 expression and purification

GST-tagged TRAF3, His-tagged full-length human HECTD3, His-tagged DOC domain (amino acid residues 219–397) and HECT domain (amino acid residues 512–857) were cloned as a *Bam*HI *Xho*I fragment into pGEX-4T-1 or pet32a (Invitrogen, Shanghai, China) and expressed as a fusion in *Escherichia. Coli* BL21 cells. The cells were cultured at 37 °C with shaking until logarithmic phase and isopropyl β-d-thiogalactoside (IPTG) was added to a final concentration of 0.1 mM. Bacteria were collected by centrifuging after 16 h shaking at 16 °C. GST-tagged TRAF3 was purified using GSH Purose 4 Fast Flow beads (Qianchun Bio, Yancheng, China) in 10 mM PBS (pH 8.0) containing 10% glycerol and eluted using 50 mM Tris-HCl (pH 8.0) containing 20 mM glutathione. The His-tagged HECTD3, His-tagged DOC, and His-tagged HECT domains were purified using Ni-NTA Purose 6 Fast Flow beads (Qianchun Bio, Yancheng, China) in 10 mM PBS (pH 8.0) containing 10% glycerol and eluted with 10 mM PBS (pH 8.0) containing 500 mM imidazole.

### GST pull-down assay

GST and GST-TRAF3 were constructed using *Escherichia. Coli* BL21 cells and were purified with GSH beads. The His-tagged full-length HECTD3 fusion protein and His-tagged DOC and HECT domains were expressed in BL21 (DE3) cells, purified, and collected with Ni-NTA beads. They were rotated with GST and GST-TRAF3 at 4 °C for 16 h and added to GSH Purose 4 Fast Flow beads (Qianchun Bio, Yancheng, China) for an additional 4 h at 4 °C. After centrifugation and three washes, the beads were eluted with 50 μL of 1× sodium dodecyl sulfate polyacrylamide gel electrophoresis (SDS-PAGE) loading buffer and boiled for 5 min, followed by western blot.

### Cell viability assay

The tetrazolium salt CCK-8 (Bimake, China) was used to determine cell viability according to the manufacturer’s protocol. BRL-3A cells were cultured in 96-well plates. After pre-treatment, CCK-8 was added to the cell culture medium at 1:10 and sequentially incubated at 37 °C for 1 h. Finally, absorbance at 450 nm was measured using a microplate reader (Molecular Devices, CA, USA).

### ROS staining

The ROS assay kit (Beyotime, Shanghai, China) was used to quantify intracellular ROS levels. BRL-3A cells were cultured in 24-well plates. After cell transfection and modeling, BRL-3A cells were washed thrice with PBS. Then, they were loaded with DCFH-DA (10 μM) for 20 min at 37 °C. To fully remove the superfluous DCFH-DA probe, BRL-3A cells were washed thrice with PBS, fixed in 4% paraformaldehyde for 20 min at room temperature, and washed three times with PBS. The cells were then observed and imaged using a fluorescence microscope (Olympus, Tokyo, Japan).

### TMA analysis

TMA was constructed from paraformaldehyde-fixed and paraffin-embedded specimens. Briefly, core human liver tissue specimens (diameter: 3 mm) were collected from typical regions of the individual donor blocks and accurately arrayed into the recipient paraffin block (45 × 22 mm) using a custom-built instrument (Beecher Instruments, Silver Spring, MD, USA). Then, the recipient paraffin block was incubated at 62 °C for 1 h, and the surface of the block was smoothed. Consecutive sections (5 μm) of the TMA block were cut with a microtome. The TMA blocks were then subjected to H&E and IHC staining.

### Human tissue specimens

Human liver samples were collected from the Zhongnan Hospital of Wuhan University. (Wuhan, China). Ethical approval was obtained from Medical Ethics Committee of Zhongnan Hospital of Wuhan University (approval number 2020122). Informed consent was obtained from all participants.

### Statistical analysis

All experimental data are shown as the mean ± standard deviation. Groups were compared using Student’s two-tailed unpaired *t*-test, one or two-way ANOVA followed by Tukey’s post-hoc test. Data analysis was performed using GraphPad Prism 8.0 (GraphPad Prism Software, La Jolla, CA, USA). *P* < 0.05 was considered significant.

## Supplementary information

Supplementary fig. 1

Supplementary table 1
