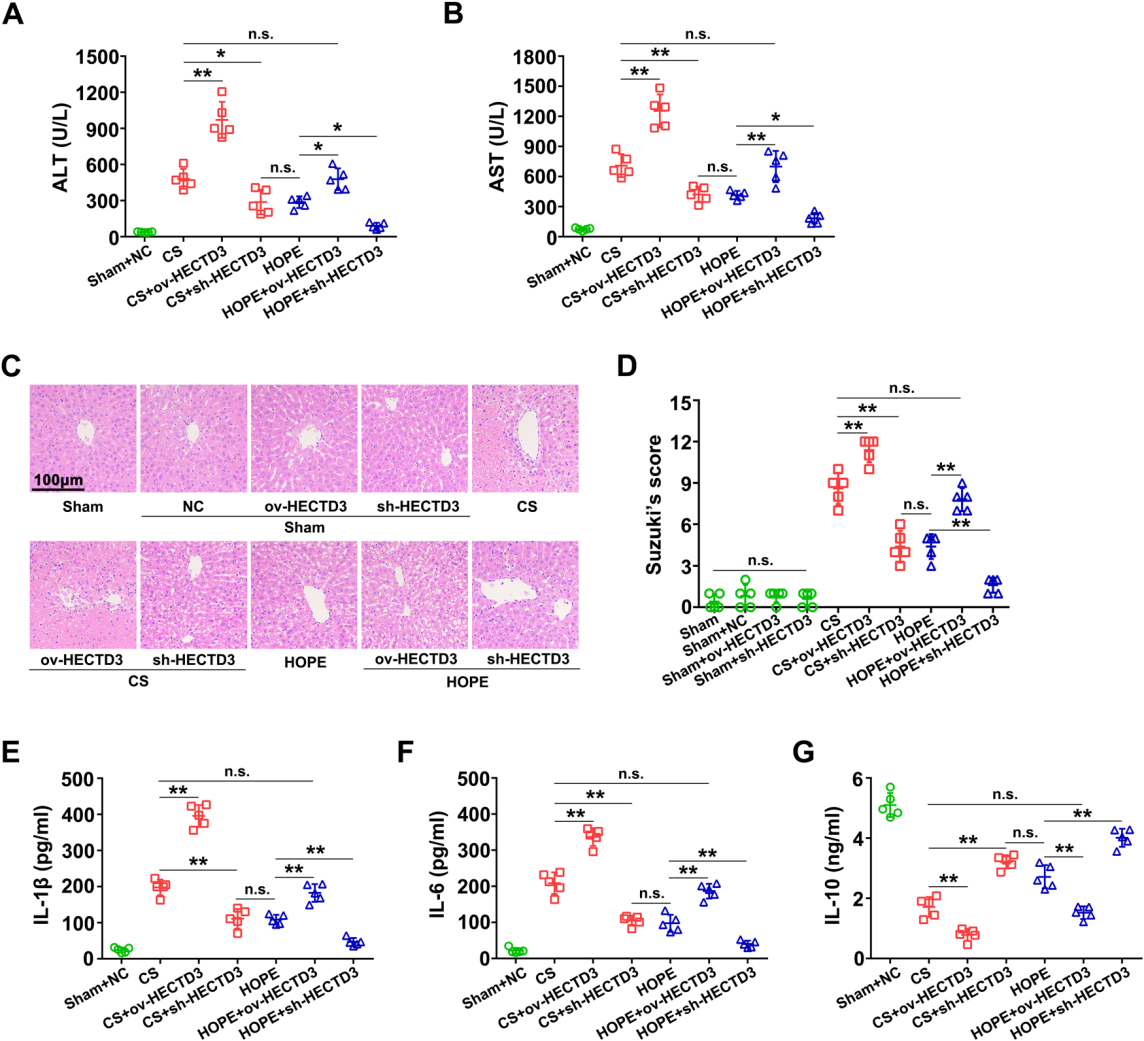# Correction: Hypothermic oxygenated perfusion inhibits HECTD3-mediated TRAF3 polyubiquitination to alleviate DCD liver ischemia-reperfusion injury

**DOI:** 10.1038/s41419-021-03840-3

**Published:** 2021-06-02

**Authors:** Wei Zhou, Zibiao Zhong, Danni Lin, Zhongzhong Liu, Qiuyan Zhang, Haoyang Xia, Sheng Peng, Anxiong Liu, Zhongshan Lu, Yanfeng Wang, Shaojun Ye, Qifa Ye

**Affiliations:** 1 Zhongnan Hospital of Wuhan University, Institute of Hepatobiliary Diseases of Wuhan University, Transplant Center of Wuhan University, Hubei Key Laboratory of Medical Technology on Transplantation, Engineering Research Center of Natural Polymer-based Medical Materials in Hubei Province, Wuhan, China; 2 The First Affiliated Hospital, Zhejiang University School of Medicine, Department of Hepatobiliary and Pancreatic Surgery, Zhejiang Provincial Key Laboratory of Pancreatic Disease, Innovation Center for the Study of Pancreatic Diseases, Hangzhou, China; 3 The 3rd Xiangya Hospital of Central South University, Research Center of National Health Ministry on Transplantation Medicine Engineering and Technology, Changsha, China

**Keywords:** Molecular biology, Diseases

Correction to: *Cell Death and Disease*

10.1038/s41419-021-03493-2 published online 24 February 2021

The original version of this article unfortunately contained an error in Figure 4. The H&E stained picture in the HOPE group in Figure 4 was wrong. The authors apologize for the error. The correct Figure 4 can be found below. The original article has been corrected.