# Supplementary material for: Hypothermic oxygenated perfusion inhibits HECTD3-mediated TRAF3 polyubiquitination to alleviate DCD liver ischemia-reperfusion injury
Source: Cell Death Dis. 2021 Feb 24;12(2):211. doi: 10.1038/s41419-021-03493-2 (PMC7904838; doi:10.1038/s41419-021-03493-2)
Supplement: Supplementary file 1 — Supplementary fig. 1 [file 41419_2021_3493_MOESM1_ESM.docx]

**
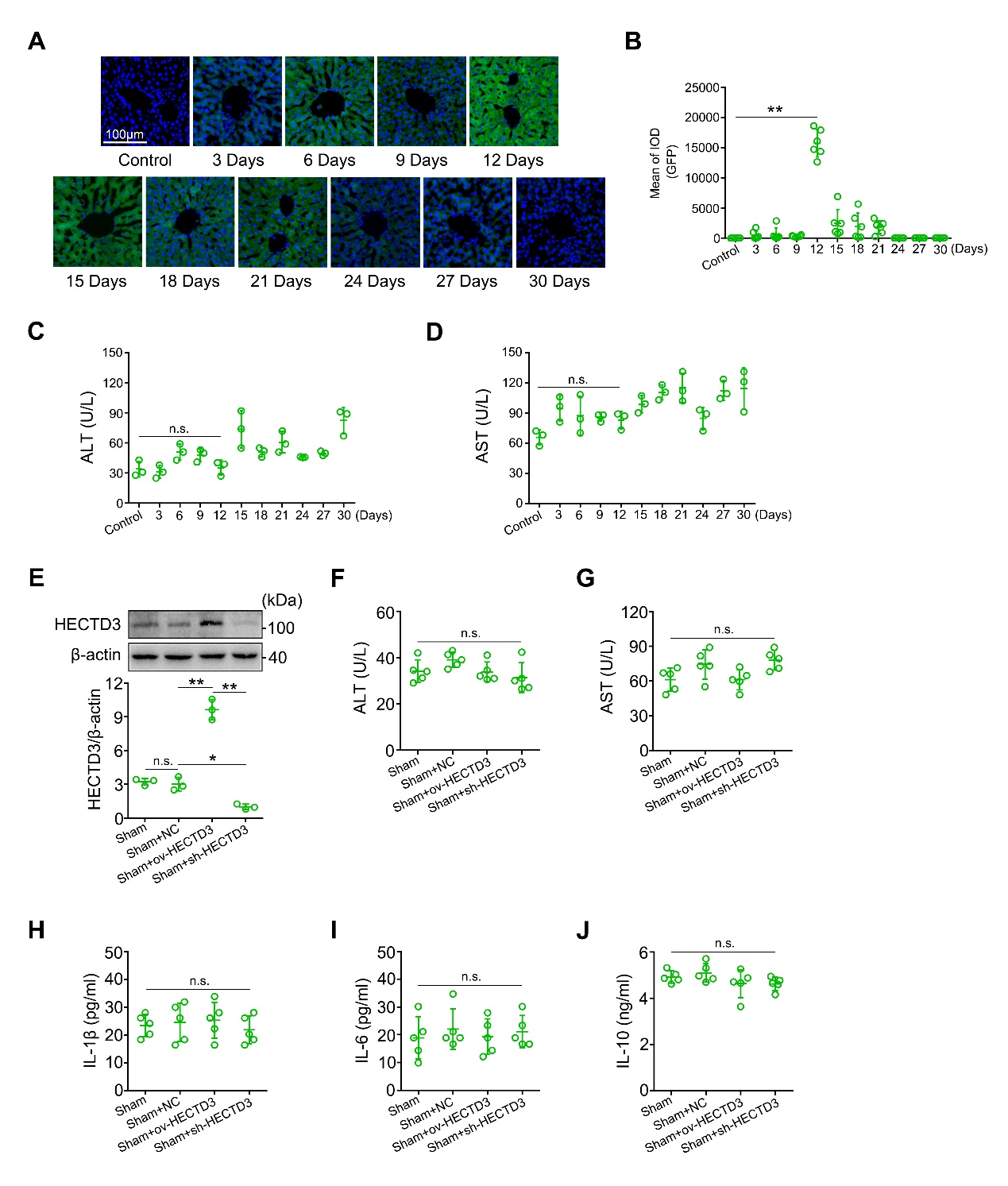
Supplemental fig. 1**

**Supplemental fig. 1** Transfection effect of HECTD3 AAV8 and its influence on liver function and inflammation. **A, B** IF staining of GFP to examine the transfection efficiency of AAV8 NC. The stained fluorescence was observed using a fluorescence microscope. Scale bar = 100 μm. IF stained fluorescence of GFP was quantified using Image-pro plus 6.0. Data are mean ± SD, ***P* < 0.01 by one-way ANOVA followed by Tukey’s test. n = 6 per group. **C, D** ALT and AST activities in rat serum. SD rats were transfected with AAV8 NC for different time. Serum was collected for ALT and AST activity measurements. Data are mean ± SD, n.s., not significant; date were analysed by one-way ANOVA followed by Tukey’s test. n = 3 per group. **E** Western blot analysis to examine the transfection effect of HECTD3 AAV8. SD rats were transfected with HECTD3 AAV8 (ov-HECTD3 or sh-HECTD3 AAV8) for 12 days. Liver tissues were collected for western blot and quantified using a Gel-Pro Analyzer. Data are mean ± SD, n.s., not significant; **P* < 0.05 and ***P* < 0.01 by one-way ANOVA followed by Tukey’s test. n = 3 per group. **F-J** SD rats were transfected with ov-HECTD3 AAV8 or sh-HECTD3 AAV8 for 12 days. After liver reperfusion, perfusate and liver tissues were collected for ALT and AST activity measurements (F, G) and the test of IL-1β, IL-6, and IL-10 using the relative ELISA kits (H-J). Data are mean ± SD, n.s., not significant; date were analysed by one-way ANOVA followed by Tukey’s test. n = 5 per group.
