# Supplementary material for: Hypothermic oxygenated perfusion inhibits HECTD3-mediated TRAF3 polyubiquitination to alleviate DCD liver ischemia-reperfusion injury
Source: Cell Death Dis. 2021 Feb 24;12(2):211. doi: 10.1038/s41419-021-03493-2 (PMC7904838; doi:10.1038/s41419-021-03493-2)
Supplement: Supplementary file 2 — Supplementary table 1 [file 41419_2021_3493_MOESM2_ESM.docx]

**Supplementary Table 1. Primer sequences of the target genes**

| **Gene name** | **Primer name** | **Primer sequences (5′-3′)** |
| --- | --- | --- |
| TNF-α | Forward  Reverse | CTGTGCCTCAGCCTCTTCTC  ACTGATGAGAGGGAGCCCAT |
| IL-1β | Forward  Reverse | AGGCTGACAGACCCCAAAAG  CTCCACGGGCAAGACATAGG |
| IL-10 | Forward  Reverse | CCTGGTAGAAGTGATGCCCC  AGACACCTTTGTCTTGGAGCTT |
| HECTD1 | Forward  Reverse | GCAGAATCCCAGGACTACGG  TATTAACGTCAGCGCCCCTC |
| HECTD2 | Forward  Reverse | AATTTGGTTCACCCACCCCT  ACCAGACTTTGCCTTGCGAT |
| HECTD3 | Forward  Reverse | GTGAGGAGAAGCTGCGCTAT  CCGTCACCTCTGTTGAGACC |
| β-actin | Forward  Reverse | ACTCTGTGTGGATTGGTGGC  CGCAGCTCAGTAACAGTCCG |
